# The Effects of Venesection and Infusion of Saline after Irritant Gas Poisoning

**Published:** 1919

**Authors:** 


**The Effects of Venesection and Infusion of Saline After Irritant Gas Poisoning.** By F. P. Underhill, S. Goldschmidt, and B. W. Wilson. *Reports of the Chemical Warfare Medical Commitee*, November, 1918.

(1) *Chlorine.* So far as the authors know, the first positive experimental results were obtained in dogs gassed with lethal concentrations of chlorine.

These results were as follows: (*a*) Effect of bleeding in dogs gassed with chlorine.

(800-900 parts per million — 1:1250 to 1:1100 for 30 minutes)

| | Acute deaths. | Delayed deaths. | Survivals. | Animals used. |
|---|---|---|---|---|
| Untreated. . | 87 o/o | 4 o/o | 9 o/o | 23 |
| Bled. . . . | 85 o/o | 5 o/o | 30 o/o | 20 |

*b*) Effect of bleeding, infusion of isotonic salt solution and administration of alkali in dogs gassed with chlorine.

(800-900 parts per million — 1:1250 to 1:1100 for 30 minutes)

| | Acute deaths. | Delayed deaths. | Survivals. | Animals used. |
|---|---|---|---|---|
| Untreated. . | 87 o/o | 4 o/o | 9 o/o | 23 |
| Treated. . . | 53 o/o | 23 o/o | 24 o/o | 62 |

It is seen from this table the latter treatment is more beneficial than bleeding alone, when applied to dogs severely gassed with chlorine.

(2) *Chlorpicrin. Method of Gassingand Technic of Experimental Methods.* The method of gassing may be outlined as follows: 10 or 12 animals were placed in the gassing chamber and left for a period of 25 minutes in an atmosphere of chlorpicrin made of one part of chlorpicrin to 8500 to 9000 parts of air. The air in the chamber was kept in motion by means of electric fan. Bleeding was performed, as fas as possible under aseptic conditions, by aspiration through a needle inserted through the skin into the right or left jugular vein.

*a*) Effect of bleeding in goats gassed with chlorpicrin.

(1:8500 to 1:9009 for 25 minutes)

| | Animals used. | Deaths. | o/o Deaths. |
|---|---|---|---|
| Untreated. . . . . . . . . . | 48 | 43 | 90 |
| Treated. . . . . . . . . . . | 47 | 27 | 57 |

*b*) Effect of bleeding in goats gassed with chlorpicrin.
Animals grouped according to time of death.

| | 3-8 hrs. | 8-24 hrs. | 24-48 hrs. | 2-7 days. | Total deaths. | Survivals. |
|---|---|---|---|---|---|---|
| Untreated. . | 19 | 18 | 2 | 4 | 43 | 5 |
| Treated. . . | 8 | 16 | 1 | 2 | 27 | 20 |

Animals surviving at beginning of period.

| | | | | |
|---|---|---|---|---|
| Untreated. . . . . . . . | 48 | 29 | 11 | 9 |
| Treated . . . . . . . . | 47 | 39 | 23 | 22 |

o/o deaths among animals surviving at beginning of period:

| | | | | |
|---|---|---|---|---|
| Untreated . . . . . . . . | 40 o/o | 62 o/o | 18 o/o | 44 o/o |
| Treated . . . . . . . . . | 17 o/o | 41 o/o | 4 o/o | 7 o/o |

Here also a greater percent. of the untreated animals die. Hence a single treatment has furnished protection to the treated animals through the severest of the acute stage of gassing. After 24 hours the number of treated animals which die is very small; on the other hand the untreated continue to give an appreciable percent. of deaths. The treatment has therefore given a permanent advantage, and has not merely delayed the death of the animal.

(3) *Phosgene.* Dogs were gassed in an atmosphere of phosgene for 30 minutes, with the following results:

*a*) Effect of bleeding in dogs gassed with phosgene.

(71-80 parts per million - 1 : 14,200 to 1 : 12,500 for 30 minutes).

The mortality in untreated animals is 78 o/o. The acute deaths are reduced from 67 o/o in untreated animals to 40 o/o in treated. The recoveries increased from 22 o/o in the untreated to 50 o/o in the treated.

*b*) Effect of multiple small bleedings in dogs gassed with phosgene at various concentrations.

In concentrations producing a mortality of about 50 o/o in the untreated animals, bleeding in small amounts at intervals shows a very decidedly beneficial effect. As the concentration increases, the beneficial effect becomes gradually less.

*c*) Effect of bleeding and early infusion in dogs gassed with phosgene.

Bleeding 1 hour after gassing to the extent of 1 o/o of the body weight, followed by an infusion of an equal quantity of isotonic sodium chloride solution, yielded results in the first 24 hours less satisfactory than the bleeding alone. Of the 50 o/o of the animals surviving the acute period, but 3 o/o died subsequently, against 10 o/o of the untreated animals.

*d*) Effect of bleeding and delayed infusion in dogs gassed with phosgene.

Here the total number of acute deaths has been reduced from 64 o/o in the untreated to 31 o/o when bled only; to 20 o/o when bled and infused with isotonic salt solution after 15-20 hours. The total recoveries are of the same order in bleeding alone and bleeding and infusion.

*e*) Effect of bleeding and delayed infusion (10 hours) in dogs gassed with phosgene at higher concentration. Here also there is an undoubted reduction of the acute deaths, especially in the very early period.

*f*) Effect of bleeding and infusion at the proper time, as indicated by hemoglobin determinations in dogs gassed with phosgene.

When the concentration of the blood is followed by the hemoglobin content and the time of infusion is properly ascertained, in addition to the great reduction of acute deaths, the percent. of total recoveries is markedly increased.

(4) *Conclusions.* *a*) Bleeding is beneficial for animals gassed with any of the 3 typical examples of the class of lung irritants: chlorine, chlorpicrin or phosgene.

*b*) Bleeding followed by accessory infusion of isotonic salt solution at a time when, by hemoglobin determinations, the blood is shown to be concentrating, and a repetition of this process at well-timed intervals increases the chances of recovery.

*c*) In so far as animal experiments may indicate, general application of these procedures to gassed men seems justifiable.

